# Dress is a Fundamental Component of Person Perception

**DOI:** 10.1177/10888683231157961

**Published:** 2023-03-23

**Authors:** Neil Hester, Eric Hehman

**Affiliations:** 1 University of Waterloo, Ontario, Canada; 2 McGill University, Montreal, Quebec, Canada

**Keywords:** clothing, fashion, style, social cognition, first impressions, social categorization, theory of mind, social status, aesthetics

## Abstract

**Academic Abstract:**

Clothing, hairstyle, makeup, and accessories influence first impressions. However, target dress is notably absent from current theories and models of person perception. We discuss three reasons for this minimal attention to dress in person perception: high theoretical complexity, incompatibility with traditional methodology, and underappreciation by the groups who have historically guided research in person perception. We propose a working model of person perception that incorporates target dress alongside target face, target body, context, and perceiver characteristics. Then, we identify four types of inferences for which perceivers rely on target dress: social categories, cognitive states, status, and aesthetics. For each of these, we review relevant work in social cognition, integrate this work with existing dress research, and propose future directions. Finally, we identify and offer solutions to the theoretical and methodological challenges accompanying the psychological study of dress.

**Public Abstract:**

Why is it that people often agonize over what to wear for a job interview, a first date, or a party? The answer is simple: They understand that others’ first impressions of them rely on their clothing, hairstyle, makeup, and accessories. Many people might be surprised, then, to learn that psychologists’ theories about how people form first impressions of others have little to say about how people dress. This is true in part because the meaning of clothing is so complex and culturally dependent. We propose a working model of first impressions that identifies four types of information that people infer from dress: people’s social identities, mental states, status, and aesthetic tastes. For each of these, we review existing research on clothing, integrate this research with related work from social psychology more broadly, and propose future directions for research.

In one of *Cinderella’s* most iconic scenes, the Fairy Godmother transforms Cinderella’s clothing from rags into a beautiful ballgown. She is now fit for the royal ball: Those in attendance greet her with awe rather than the disdain she would have faced arriving in her old, threadbare clothes. At its core, this scene is about the transformative power of dress. One outfit changes others’ impressions of Cinderella, and she lives happily ever after. Though most would agree that dress substantially influences perceptions and outcomes, this topic has received relatively little attention in social psychology. This oversight raises questions regarding the validity of person perception models that do not incorporate target dress. Here, we review existing literature, propose a working model to integrate dress with the broader impression formation literature, and identify four important ways in which dress impacts person perception.

Despite lay consensus that dress is important for first impressions, recent reviews of social categorization and evaluation processes (Bacev-Giles, & Haji, 2017; Kang & Bodenhausen, 2015; Pauker et al., 2018; Rule & Sutherland, 2017) have little to say about the impact of dress on these processes. This lack of attention contrasts sharply with heavily-researched factors involved in person perception, such as facial appearance. The role of faces in person perception has been studied for decades (Bruce & Young, 1986; Ekman & Friesen, 1971; Rhodes, 2006; Secord & Bevan, 1956; Todorov et al., 2015), with many of the most prominent theories and models of person perception (Oosterhof & Todorov, 2008; Vernon et al., 2014; Zebrowitz et al., 2003) founded on the study of faces—almost always disembodied faces absent body or clothing for purposes of experimental control. However, people in the wild regularly perceive faces in a fully embodied state—that is, with an entire outfit and body unavoidably integrated into the categorization and evaluation process. If psychologists’ goal is to form theories of person perception that explain and predict real-world judgments, then it is important to begin studying impressions of more realistic and complex stimuli.

Of course, overall impressions emerge from dress alongside numerous other factors such as emotion expression, dynamic movement, body, surrounding context, and perceivers’ beliefs and motivations. The present work focuses on the importance of dress in person perception both beyond and in conjunction with these other important factors. The importance of dress has not been altogether ignored by researchers, as there is a considerable array of research on clothing across multiple fields: psychology, sociology, anthropology, marketing, and others. However, researchers have often studied dress piecemeal and for idiosyncratic purposes, focusing on specific phenomena (e.g., associations of red with attractiveness; signals of wealth or sexual interest; see K. Johnson et al., 2014). One review, which aimed to “present a comprehensive review and analysis of published research that investigated relationships between the dress of an individual and how that dress affected others’ behavior toward the individual” (K. Johnson et al., 2008, p. 3) ends its abstract with the statement “Most of this research was not guided by theory” (p. 3). For this reason, most existing research on the psychology of dress is difficult to situate and understand within broader theoretical frameworks in person perception and social cognition.

Here, we argue that dress is an essential and underappreciated element of person perception that should be incorporated in theoretical models. We first provide a formal definition of the term “dress.” Then, we reflect on three possible reasons for the historical neglect of dress in social psychology: high complexity, incompatibility with dominant methods, and cultural association with oppressed groups. Finally, we propose a working model of person perception in which perceivers make sense of the targets’ dress, body, face, and other features through their socioperceptual “lens” (i.e., their cultural knowledge, stereotypes, and beliefs) to form and update impressions. Within the target dress part of this model of person perception, we outline four factors or signals that observers may glean from the dress of targets: social categories, cognitive states, status, and aesthetics. This list is non-exhaustive: We present this model as a tool for organizing the existing literature on dress and person perception and identifying future directions for research.

## Defining Dress

Like many categories (e.g., bird, soup), the concept of “dress” is fuzzy. People generally have an intuitive sense of what dress is, yet drawing a hard line around what is and is not dress is difficult. Typical articles of clothing—shirts and pants, socks and shoes, overcoats and undergarments—are clearly elements of dress. Biological physical characteristics of the face and body such as height, weight, muscularity, and face shape are clearly not elements of dress. Objects in the environment such as beds, rugs, and paintings are similarly not elements of dress. In these cases, what is and is not dress is uncontroversial. However, what should we make of hair and hairstyles, makeup and tattoos? If a wheelchair or prosthetic is needed for movement, is it an element of dress? What about backpacks, purses, and bags?

A formal definition of dress helps to resolve these ambiguities. Here, we draw on foundational work of Mary Ellen Roach-Higgins and Joanne Eicher (1965, 1973, 1992):

**Dress** of an individual is an assemblage of modifications of the body and/or supplements to the body. (Roach-Higgins & Eicher, 1992, p. 1)

Roach-Higgins and Eicher, (1992) describe this definition as “unambiguous, free of personal or social valuing or bias, usable in descriptions across national and cultural boundaries, and inclusive of all phenomena that can be accurately designated as dress” (p. 1). They also note that this definition does impose “a somewhat arbitrary conceptual separation between biologically determined body characteristics and dress” (p. 1), acknowledging the conceptual fuzziness between body and dress. A detailed account of the term “dress” versus related terms (e.g., clothing, appearance, adornment) can be found in Roach-Higgins and Eicher (1992).

As we are primarily concerned with dress in the context of person perception, it is useful to note that *target dress*, as understood through the eyes of a perceiver, is not fully inclusive of *dress* as it is experienced by the target individual. There are modifications and supplements of the body that are invisible to some perceivers because they are underneath clothing (e.g., tattoos), appear to occur naturally (e.g., plastic surgery), or are otherwise imperceptible (e.g., some perceivers have color blindness).

With this definition in mind, we can reflect on the aforementioned cases. Hair itself is not dress; however, styling one’s hair is, as doing so modifies the body. Makeup and tattoos are dress, despite not being as explicitly “material” as clothing. Physical aids such as wheelchairs and prosthetics present a trickier case; although they do modify the body, they do so in a way that is meant to restore and extend functioning, similar to a tool, making their status as elements of “dress” unclear, though specific colors or styles of physical aids may more clearly be seen as dress. Backpacks and purses are typically chosen to supplement the body’s carrying capacity and also modify the body’s appearance; grocery bags, though similar, perhaps are not experienced or perceived as “extensions” of the body in the same way.

This definition of dress is by no means perfect, and readers may disagree with our descriptions of these fringe cases or highlight cases that seem odd to label as “dress” (e.g., the visual results of dental surgery, or scars from an accident). However, this exercise draws some useful boundaries around “dress” as a key concept in person perception. In the next section, we highlight the dearth of theory describing the role of dress in person perception.

## The Neglect of Dress in Person Perception

At a glance, dress seems to strongly influence impressions of others, guiding perceivers’ inferences about targets’ personalities, interests, and status. What, then, might explain the lack of research and theory on dress in the person perception literature? We discuss three potential reasons: high complexity, incompatibility with traditional methodology, and cultural associations with oppressed groups.

### High Complexity, Emergent Properties

Assembling a “good” outfit is an undeniably pressing task for some people (including one author of the present work) because it requires combining several discrete items of clothing into a single, harmonious whole—a Gestalt of style. Outfits present a similarly vexing issue for psychologists who wish to form theory or inferences about dress. The impact of a given item of clothing on perceptions of any individual cannot be understood in a vacuum: the same pair of jeans could elicit wildly different impressions depending on what it’s worn with, who’s wearing it (their face and body type), where they’re wearing it, and when (the season, the century)—not to mention the idiosyncrasies of the perceivers themselves. Furthermore, the range of variability in facial features (e.g., eye size, face length) and body features (e.g., arm length, waist-to-hip ratio) are restricted by biology and thus fairly similar across cultures, whereas dress has no such biological restriction of range.

Furthermore, because the psychological meaning of dress is socially and culturally constructed, perhaps to an even greater degree than many other psychological factors (given its proximity to art and aesthetics; Miller, 2007), research on dress is less compatible with the goal of uncovering “psychological universals”—a goal that is often assigned high theoretical value. For example, in research on person perception, evolutionary theories have had an outsized impact in part because evolutionary perspectives align with the idea of psychological universals. Research on perceived attractiveness relies on the idea that sexual selection prioritizes sexually dimorphic traits (Perrett et al., 1998; Thornhill & Gangestad, 1999), and the valence-dominance model of face perception identified Dominance and Trustworthiness/Valence because of the evolutionary functions served by inferring these traits from faces (Oosterhof & Todorov, 2008; Vernon et al., 2014). These parsimonious and universalist accounts have since undergone revisions: sexual dimorphism does a poor job accounting for gendered face perception (Hester, Jones, & Hehman, 2021), and the dimensions that describe face perception vary across cultures and social groups (Jones et al., 2021; Xie et al., 2021).

These revisions reflect an ongoing value shift in social psychology, which as a field increasingly embraces complexity, incorporates cultural variability, and tests the generalizability of well-established models. However, this shift also poses practical challenges for designing studies and analyzing data. Many of the traditional methods in social psychology are ill-suited for handling highly complex phenomena, which is another possible contributor to the minimal study of dress in person perception.

### Incompatibility With Traditional Methodology

Experimentation is the main approach used by social psychologists for causal inference. Some researchers have argued that this heavy focus on experimentation can be attributed to modeling psychology after more mature sciences such as biology and chemistry (Oishi et al., 2009; Rozin, 2001). In this argument, the 2 × 2 experiment—simple enough to calculate statistics for by hand, complex enough to reveal exciting contextual factors—became the gold standard, which led Moin Syed (2021) to quip that social psychology is “2 × 2 designs all the way down.” However, this methodology constrains the universe of questions that can be asked, such that researchers may have been encouraged to test hypotheses that were more amenable to being answered by 2 × 2 experimental designs.

Due to its high complexity, dress is not amenable to being researched in 2 × 2 experimental designs. Studies in this traditional framework would either lack generalizability (studying only a narrow array of dress options) or quickly sprawl out of control (e.g., a 10 × 5 × 8 design). Furthermore, the creation of tightly controlled stimuli would have posed a nigh insurmountable methodological challenge. Any given article of clothing varies in color, fit, fabric, and pattern; and, it influences impressions in tandem with other articles of clothing, the body of the wearer, and various other factors. Although researchers now embrace and model heterogeneity in stimuli to a greater extent, historically this has not been the case (Judd et al., 2012; Westfall et al., 2015).

This greater embrace of heterogeneity aligns with a broader emphasis on external validity, which requires testing psychological phenomena across a more representative population of participants, targets, and contexts. This brings us to one more potential reason why dress may have been neglected in the study of person perception: The people who best understand dress as part of their lived experience have not, historically, been those conducting and guiding research on impressions.

### Dress as Expression and Communication for Oppressed Groups

In the past couple of decades, social psychology has reckoned with a “crisis of generalizability.” The field’s overreliance on samples from WEIRD populations became readily apparent (Henrich et al., 2010), and intersectionality scholars highlighted the absence of myriad multidimensional groups from most psychological work (Cole, 2009; Goff & Kahn, 2013; McCormick-Huhn et al., 2019; Settles et al., 2020). Many of the majority-group researchers who have made universal claims likely view the world through their majority-group lenses, leading them to misperceive the importance of various phenomena to human experience as a whole.

To be sure, individuals across the entire spectrum of status care about dress. Red carpet events and high fashion brands highlight the sartorial tastes of wealthy and powerful individuals. However, those with power have the resources to express themselves in a wide variety of ways, whereas those lacking power have fewer means of expression. Dress is perhaps one of the primary vectors through which individuals lower in power and status can express themselves. Don Letts, a prominent DJ and filmmaker, echoed this sentiment: “You’ve got to understand. Black, working-class kid, that’s the only way we had to express ourselves was through the music we listened to and the clothes we wore” (Trufelman, 2018, pp. 3:05–3:15).

Thus, the importance of dress as a factor in person perception might be most immediately understood by members of groups that have been historically excluded from the powerful, piloting positions of the field—people who cannot afford to wear certain kinds of clothes (e.g., poor people), who face strict cultural expectations about appropriate clothing (e.g., women), and who sometimes feel pressure to wear clothes that do not match their felt identity (e.g., LBGTQ+ [lesbian, gay, bisexual, transgender, queer/questioning, plus (others)] people). In her podcast series on dress and history, Avery Trufelman addresses the myth of fashion as frivolity:

There is this myth, that it’s frivolous or unproductive to care about how you look. Clothing and fashion get trivialized a lot. But think about who, culturally, gets associated with clothing and fashion: young people, women, queers, and people of color. Groups of people who, historically, haven’t been listened to, have expressed themselves on their bodies, through their style, their hair, their tattoos, their piercings, and what they wear. (Trufelman, 2018, pp. 2:40–3:05)

In addition to being a form of self-expression, dress is also a vital identity-signaling tool for oppressed groups. For example, across history, members of the LGBTQ+ community have used dress to subtly but clearly state their sexual orientation and gender identity to other members of the community, allowing relationships and networks to form without being “out” to the general public (Clarke, 2013).

In this way, it seems probable that the historical dominance of social psychology by researchers who are mostly White (S. O. Roberts et al., 2020), male (Gruber et al., 2021), and from educated and wealthy backgrounds (Morgan et al., 2021) contributes to the dearth of research on the psychology of dress. Critical scholarly work on fashion has better incorporated ideas from intersectionality theory and LBGTQ+ discourse (e.g., see Pritchard, 2017 for an introduction to a full journal issue on “sartorial politics, intersectionality, and queer worldmaking”), and psychologists might look to this work for new ideas and theoretical guidance. In the next section, we will see that existing work on the psychology of dress—construed broadly and drawing from several adjacent fields—has not yet been organized by a central theoretical model.

## The Disparate Phenomena of Dress

Although psychological research on dress is not organized by a central theoretical model, various pockets of research have emerged across psychology and adjacent fields. K. Johnson and colleagues (2014) provide a useful review of these pockets, which we briefly summarize. One pocket of research explores perceivers’ inferences about women’s sexual interest based on their outfits (e.g., Farris et al., 2008; Mathes & Kempher, 1976; Montemurro & Gillen, 2013; Perilloux et al., 2012). Another, inspired by evolutionary theories of sexual selection, specifically considers the impact of the color red on perceived attractiveness (Elliot et al., 2013; Kayser et al., 2010). Yet another pocket of research focuses instead on the influence of dress on the wearer’s cognition (e.g., enclothed cognition; Adam & Galinsky, 2012; Frank & Gilovich, 1988; Fredrickson et al., 1998).

Some research, often located in business journals, has measured how signals of wealth (e.g., a Timex versus a Rolex) influence judgments of targets (Maaravi & Hameiri, 2019; Shutts et al., 2016). Another pocket of research, regularly found in applied professional journals, describes the effect of different types of dress on perceptions of professionalism and competence (e.g., street clothes vs. a blue medical coat vs. a white medical coat for nurse; Albert et al., 2008; Furnham et al., 2013) as they relate to outcomes such as job evaluations and hiring decisions. In personality psychology, work investigates the extent to which clothing (a) accurately signals wearers’ personality and (b) is used by perceivers to form impressions (Gosling et al., 2002; Naumann et al., 2009). Finally, there is a smattering of work on how dress influences social categorization—that is, categorizing someone based on articles of clothing or accessories (e.g., lesbians and carabiners; conservatives and pearl earrings; punk rockers and safety pins; Carroll & Gilroy, 2002; Clarke, 2013; Hayfield, 2013).

Our intention in this section is not to provide a fully exhaustive review of literature on dress in psychology (for reviews, see K. Johnson et al., 2008, 2014; Lennon et al., 2017, 2014) but instead to highlight that dress research is largely restricted to tests of specific phenomena, such that there are currently no unified theoretical models describing how dress affects impressions. In the next section, we draw on existing theories and knowledge in person perception to propose an initial theoretical model of impressions that describes how they emerge from the combination of target face/body, target dress, and target context, as understood through the perceivers’ lens (e.g., their beliefs, stereotypes, attitudes, and preferences). Then, we will identify four factors of target dress in person perception to provide a framework within which we can organize past research and identify questions for future research.

## Incorporating Dress Into Models of Impressions

Before discussing the specific facets of dress, it is important to place dress within a general model of person perception. Our model follows the theoretical statements made by the Dynamic Interactive model of person perception (Freeman & Ambady, 2011; Freeman et al., 2020), in which emerging impressions of one feature of a target’s identity (e.g., their skin color) excite and constrain other connected features in a dynamic network. We distinguish our model from previous models of person perception by recognizing target dress as a feature of target identity that is sufficiently distinct from target face/body (which we collapse into one “factor” here) to merit its own separate consideration. Target face/body and target dress are markedly different. Target face/body is more stable, trait-like, and resistant to change. As such, the causal relation between targets’ cognition or identity and their face/body is weak to nonexistent. On the other hand, target dress is more state-like and malleable. As such, the causal relation between targets’ cognition or identity and their dress is likely stronger. In addition, target face/body and dress are separate but intimately linked. Target face/body causally influences target dress (i.e., people actively choose clothing to “amplify, downplay, and sometimes defy” aspects of the body; Daniels, 2021, p. 358), and target dress is almost inevitably interpreted in conjunction with target face/body. We also include target context as another feature that influences perceivers’ judgments—that is, where the target is located, who they are with, what they are doing, and anything else that is external to both their body and their outfit.

**Figure 1.**
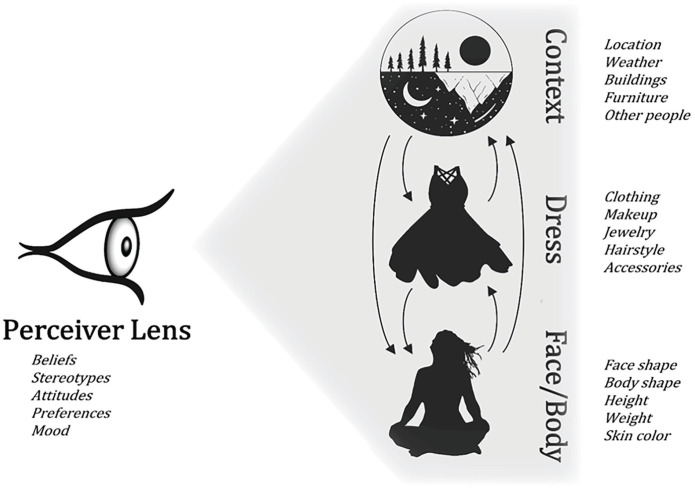
Model of Person Perception Incorporating Target Dress. *Note.* The perceiver makes sense of the target by simultaneously integrating the target’s dress, face/body, and context. This is done through the perceiver’s lens, which includes their cultural knowledge, stereotypes, attitudes, and preferences.

Figure 1 depicts a working model of person perception in which perceivers dynamically and interactively form an impression of the target, simultaneously incorporating information about face/body, dress, and context. Perceivers make sense of this information through their own perceptual lens, incorporating their individual beliefs, stereotypes, attitudes, and preferences. To unpack “dynamically and interactively” more concretely, we refer to recent work by Freeman and colleagues (2020), which describes initial social perceptions as the “rapid, yet gradual, process of negotiation between the multiple visual features inherent to a person . . . and the baggage a perceiver brings to the perceptual process” (p. 3).

For example, imagine a target wearing a generally nondescript outfit but with safety pin accessories. One perceiver might have the cultural knowledge needed to associate wearing safety pins as signals of punk identity (Rutten & van Dienderen, 2013). Because they hold positive attitudes toward people who identify as “punk,” they form a positive first impression of the target. However, another perceiver might not have this cultural knowledge, failing to identify this target as “punk.” This perceiver endorses negative stereotypes about the punk identity, but because they failed to identify this person as punk, these negative stereotypes never come into play and the perceiver relies on other cues to judge the target.

Another example highlights the interplay between target dress, target face/body, and target context. Imagine a perceiver who endorses negative Black stereotypes. In the context of an empty street at night, this perceiver might perceive a Black man (race identified by his skin color and face) wearing a hoodie as considerably more threatening than the same Black man wearing a suit and tie *or* a White man wearing either outfit. However, in the context of a restaurant in the middle of the day, the same perceiver might perceive similarly low threat in both the Black man wearing the hoodie and the same Black man wearing the suit and tie. The effect of context might be conceptualized in various ways (context could shift perceivers’ attention to threat, or maybe their Black threat stereotype is specifically about Black men in hoodies at night), but context undoubtedly moderates the interplay between perceiver lens, target face/body, and target dress.

The next section of the article will consider four specific factors of target dress, which describe the kinds of information perceivers infer from target dress. Throughout this next section, our proposed working model of person perception will be used to consider future research directions.

## Factors of Dress in Person Perception

For this working model of person perception, we propose four factors within target dress: social categories, cognitive states, status, and aesthetics. These factors describe the types of inferences that perceivers make about targets based on targets’ dress. We clearly acknowledge that these four factors are not exhaustive, nor were they empirically derived: Instead, they are themes that emerged during the literature review that parsimoniously organize most of the existing literature on dress in person perception while also possessing clear links to non-dress research in social cognition. Notably, these four factors of dress emphasize inferences that are sometimes more readily conveyed by dress than by faces, bodies, or context. In other words, target dress covers different areas of the “space” underlying impression formation than other target factors—areas that, in some cases, may hardly be tapped in the absence of information about target dress. We consider this a useful organizing framework for existing literature and for future research, even if other researchers prefer alternative or additional groupings.

For each of these four factors, we will define and describe it; connect the factor with non-dress research in person perception and social cognition; identify existing dress research that fits within the factor; and describe future directions for research.

### Social Categories

“The beltside key ring is one of the most enduring sartorial symbols of lesbian culture, one of the few stereotypes of our kind that’s both inoffensive and true” (Cauterucci, 2016). The key ring has served as a reliable signal of lesbian identity in North America for the last several decades. Like the key ring, myriad visual markers of sexual identity and gender expression exist across cultures and time periods with the express purpose of identity signaling. In cultures for which non-normative identities lead to personal danger, these signals can be quite subtle and may require “insider” cultural knowledge to accurately detect. These subtle signals have been defined by sociologist Michael Brake as “argot,” the hidden meaning carried within articles of dress (Brake, 1985/2013). However, whether signals are subtle or blatant, perceivers with the cultural knowledge to understand signals use them to make inferences about the social identity of the target.

The *social categories* factor describes how perceivers use information provided by dress to identify targets with specific social categories, including (but not limited to) sexual preference, gender identity, race, ethnicity, nationality, religious group, and political affiliation. This also includes more specific identities associated with specific cultural groups or activities, such as identification with artistic or musical movements (e.g., reggae), sports or sports teams (e.g., Tarheels fan), social archetypes (e.g., emo), and occupations (e.g., firefighter). The questions perceivers are answering in this factor include “Who is this person?” “What groups does this person identify with?” and “What does this person believe?”

#### Connections to Social Cognition Research

Social categorization is a key topic in person perception. Modern theories such as the Dynamic Interactive model of person perception (Freeman & Ambady, 2011; Freeman et al., 2020) posit that social categorization is a dynamic process in which various inputs—both bottom-up features of the face and body as well as top-down features such as perceivers’ stereotypes—interact with each other over time until a stable category judgment is reached. Some research has revealed that the clothing of targets does change the ease with which targets can be socially categorized, as non-stereotypical clothing activates competing response categories during the process of categorization (Freeman et al., 2011). Although a recent review of this dynamic model (Freeman et al., 2020) acknowledges the role of clothing and hairstyle (both aspects of dress) as bottom-up cues of social category, research on these cues—clothing in particular—is sparse.

The omission of dress from much of this work is problematic because dress may be highly relevant to the accuracy of categorization judgments. Perceivers who rely on facial information alone show high accuracy in judgments of some social categories (e.g., age, gender, race) and slightly “above chance” accuracy in judgments of more ambiguous social categories (e.g., sexual orientation, political and religious affiliation; Rule & Sutherland, 2017). However, unlike faces and bodies, dress is consciously chosen, sometimes specifically for its ability to signal social identity or for its value for performing one’s identity (e.g., one’s gendered or sexual self; see Morgenroth & Ryan, 2021). If a woman is wearing short hair, dungarees, lace-up boots, and a ring of keys (as in the Broadway song “Ring of Keys”; Tesori, 2013), this choice is intentional and more likely to be signal than noise. Thus, if perceivers were able to incorporate target dress alongside target faces, they might achieve higher accuracy when guessing others’ sexual orientation and religious affiliations, as well as aspects of personality that might also be thought of as “categories,” such as introversion/extroversion.

Even when dress is not chosen with high intentionality, it still contains residual information that can sometimes promote accurate judgments. Previous work has demonstrated that people’s inhabited environments (e.g., bedrooms, offices) contain various cues (e.g., a snowboard in the corner, a highly organized desk) that perceivers use to infer behaviors (e.g., snowboarding, organizing) that can then be linked to underlying personality traits (e.g., sensation seeking, conscientiousness; Gosling et al., 2002). Or, in other cases, perceivers might use “identity claims” (e.g., a cross or a rosary) to infer social categories (e.g., Christian), which then activate personality trait judgments via stereotyping. The accuracy of these cues varies depending on the specific trait, but some aspects of personality, such as openness to experience, can be accurately conveyed by these residual cues (Gosling et al., 2002).

#### Integration of Existing Dress Research

Much of the research on dress and social categorization has focused on sexual preference, and even this literature is somewhat thin (Clarke, 2013). Although ingroup perceivers have more intimate knowledge of dress cues for gay and lesbian sexual preferences, heterosexual university students also reported strong knowledge of the same cues, but referred to these cues as stereotypes and expressed reluctance to endorse these cues as meaningful signals (Hayfield, 2013). This is in contrast to lesbians and gay men surveyed about the usefulness of various cues for accurately identifying ingroup members, who readily identified clothing style, clothing fit, and jewelry as cues that increase categorization accuracy (Carroll & Gilroy, 2002).

Interestingly, many Western lesbians and gay men report feeling some degree of pressure to adopt the dress associated with their identities, creating tension between “sub-cultural authenticity” (dressing in a way that is clearly non-heterosexual) and “individual authenticity” (dressing based on personal tastes; Clarke & Spence, 2013). This “coercive element” (quoted in Clarke, 2013, p. 3; Levitt et al., 2003; Y. Taylor, 2007) of dress and sexual identity may actually increase the accuracy of these cues. However, there is some evidence that this coercive element is weakening in younger generations (e.g., Wilkinson, 2015), which might lead to a subsequent decrease in the accuracy of certain cues, possibly due to changing attitudes toward the LGBTQ+ community in the Western world (Charlesworth & Banaji, 2019). Conversely, historical events can lead to dramatic increases or decreases in social identity signaling (e.g., women and compulsory hijabs in Iran following the Iranian Revolution; BBC, 2019).

Prior work has also examined the extent to which specific clothing cues—stylish versus unstylish, distinctive versus ordinary, neat versus messy—both (a) actually correspond with targets’ personalities (cue validity) and (b) are utilized by observers to make judgments (cue utilization; Naumann et al., 2009; Vazire et al., 2008). Some high-validity cues were utilized (e.g., distinctive appearance for openness to experience); some high-validity cues were not utilized (e.g., stylish appearance and extraversion); and some low-validity cues were nevertheless utilized (e.g., neat appearance and conscientiousness). Thus, this work highlights how heterogeneous both the validity and utilization of dress cues might be for inferring personality traits specifically (and, likely, ambiguous social categories more broadly).

#### Future Directions

Perceivers often rely on target dress as a source of information to infer targets’ identity, and these inferences in turn can lead to the application of cultural stereotypes. Furthermore, because targets choose their dress more than their face and body, inferences based on target dress are likely to be more accurate, though this varies to the extent that the dress is intended to signal identity. With these observations in mind, researchers might consider the extent to which perceivers’ categorization of targets on various dimensions of identity relies on target dress versus target face/body, using a descriptive variance-partitioning method (Hehman et al., 2017; Hester, Xie, & Hehman, 2021; Xie et al., 2019). It might also be the case that some elements of dress (e.g., shoes; Gillath et al., 2012) provide more information about certain dimensions of identity than other elements of dress.

Researchers might also measure the extent to which target dress versus target face/body both lead to accurate judgments of identity across these various dimensions and are actually utilized by observers (Gosling et al., 2002; Naumann et al., 2009). Relatedly, one might measure the relation between perceivers’ negative stereotypes of certain groups and their cultural knowledge of specific dress-related cues. In other words, how good are perceivers at accurately identifying the groups toward which they are prejudiced? How common are false positive or false negative judgments? Furthermore, are perceivers who have positive attitudes toward certain groups knowledgeable about dress-related cues but reluctant to use them for fear of being prejudiced?

In addition, longitudinal work might examine how cultural events change the strength of “coercion” for people with certain identities to wear certain kinds of clothing, and how this coercion subsequently influences the accuracy of perceiver judgments that rely on these aspects of target dress. Given the challenges of conducting this work with self-report information, it might be necessary to adopt novel strategies for studying cultural evolution, such as coding and analyzing variables over time using written and visual media (e.g., fashion magazines; see Kuipers et al., 2017; van der Laan & Kuipers, 2016) and creating quantitative ethnographic records (Watts et al., 2022).

### Cognitive States

It’s like armor to me. When I have a suit on I feel like all of a sudden, the world sees me differently. Cops aren’t staring, people wave back, people shake my hand, they open the door for me. It’s like I’m the president of the United States. (Yi, 2015)

In David Yi’s story on “Black Armor,” Alex Peay reflects on the bodily safety and confidence bestowed by formalwear. For Black men in the United States, police officers represent the persistent fear of being stopped, questioned, arrested, or harmed. These disparities may be partly rooted in police officers’ perceptions of Black men as threatening—that is, holding the intent to harm others or commit crimes (Goff et al., 2014; Hester & Gray, 2018). Formalwear actively signals the *absence* of threatening intent, enabling Black men to navigate their environment more comfortably.

The *cognitive states* factor describes how perceivers use information provided by dress to make inferences about target cognition. In contrast with social categorization, which concerns a trait-level aspect of person perception, theory of mind concerns a state-level aspect of person perception—mental states such as goals and intentions fluctuate over time. The mental states perceivers might infer from dress include a person’s goals or motives, which could be either broadly defined (e.g., finding a sexual partner, inflicting harm on someone) or quite specific (e.g., attending a wedding, skipping school). The questions perceivers are answering in this factor include “What does this person want?” “What is this person doing?” and “Where is this person going?”

#### Connections to Social Cognition Research

One obvious connection spanning social, developmental, and clinical psychology is research on theory of mind, which describes perceivers’ ability to infer others’ mental states. The information used by perceivers for theory of mind include situational cues (e.g., the “Sally-Anne” ball-hiding task; Baron-Cohen et al., 1985), emotion expression in the face and eyes (Baron-Cohen et al., 2001; Fang et al., 2018; Montepare & Dobish, 2003), and body posture (Abele & Yzerbyt, 2021; Dael et al., 2012). As theory of mind is an essential social-cognitive process for navigating the world, its early developmental trajectory has been thoroughly mapped (Astington & Jenkins, 1995; Shahaeian et al., 2011).

In the early days of social psychology, role theory played a dominant part in researchers’ understanding of attitudes and behaviors. Although a full review of role theory is beyond the scope of this article, roles are generally defined as social scripts a person is expected to follow (either prescriptively or based on perceivers’ own beliefs about the role; Biddle, 1986). For example, if a person is identified as a police officer, then their perceived role might be to protect the populous (as culturally prescribed) or protect the status of the majority group (as believed by some).

Recent work has proposed a “Relevance Appraisal Matrix,” in which perceivers infer to what extent a person represents opportunity and threat on distinct dimensions based on current goals (Lassetter et al., 2021; Neel & Lassetter, 2019). This relevance incorporates both the current context and perceptions of the face/body to make inferences about both the stable traits and the current mental state of targets—key information for deciding whether the target is relevant to goals such as “avoid physical harm” or “find a sexual partner.” Target dress undoubtedly plays a role in these mental state inferences, especially given the bodies of research specifically investigating biases in perceptions of threat (Baumann & DeSteno, 2010; Green & Phillips, 2004; Todd et al., 2016) and biases in men’s perceptions of women’s sexual interest (Perilloux et al., 2012; Treat et al., 2015).

#### Integration of Existing Dress Research

One long-enduring line of dress research investigates how clothing sometimes indicates a specific role in society—that is, a person’s occupation, the functions that they are expected to serve, and the specific behaviors expected from them. Leslie Davis (1984) provides a useful review of early dress research relevant to role theory. Highlights from her review include uniforms strongly defining roles and leading perceivers to expect certain behaviors of target (Joseph & Alex, 1972); street clothes causing nurses to receive more positive responses from many patients because of negative beliefs about nurses in uniforms (Rinn, 1976); and non-militaristic police officer uniforms leading perceivers to have more positive expectations of and interactions with officers (Tenzel & Cizanckas, 1973).

In some cases, occupations or contexts are coded to various social groups as well, such that perceivers associate the “correct” disposition with signals of Whiteness, maleness, or other identities. For example, Black people—especially Black women—experience pressure to conform to Eurocentric hairstyles when working in professional environments (Dawson et al., 2019; Rosette & Dumas, 2007), with Afrocentric hairstyles eliciting lower ratings of professionalism for Black women (A. M. Johnson et al., 2017; Koval & Rosette, 2021; Opie & Phillips, 2015). Given the high personal, social, and financial costs of maintaining Eurocentric hairstyles, professional Black women face an ongoing double bind with regard to their hair. Similarly, women in male-dominated occupations manage the balancing act of adopting conventional male attire to belong and signal competence while still maintaining femininity and attractiveness (see S. K. Johnson et al., 2010; Sheppard & Johnson, 2019).

Another line of research focuses on how women’s dress influences perceivers’ judgments of their sexual interest. This work, in line with other heteronormative trends in dress research, focuses on straight Western male reactions to female targets. Researchers have found that perceivers judged female targets wearing sexually provocative dress as more attractive, sexually appealing, and interested in sexual acts, but also judged these female targets as more likely to cheat in relationships and use sex to manipulate men for gain (Abbey et al., 1987; K. Johnson & Workman, 1992; Mathes & Kempher, 1976; Maurer & Robinson, 2008; Montemurro & Gillen, 2013). Relatedly, other research concerns the “sexiness” or formality (manipulated via combinations of color, fit, and cut) of women’s dress and consequences in various professional settings, such as job interviews (Forsythe, 1990; K. Johnson & Roach-Higgins, 1987), classical music auditions (Griffiths, 2008, 2010; Urbaniak & Mitchell, 2022), and university lectures (Lukavsky et al., 1995; Morris et al., 1996). Again, research in this area has largely focused on impressions of women specifically (with exceptions; see Furnham et al., 2013; Slabbert, 2019).

#### Future Directions

The extent to which perceivers incorporate dress into inferences about cognitive states depends both on the target’s dress and the perceiver’s beliefs about that dress. Researchers might examine perceivers’ cultural knowledge and beliefs about various types of dress. For example, some perceivers might associate hoodies or chains with intention to harm or short skirts and heels with intention to engage in sexual acts, while other perceivers might not. These patterns of belief likely correspond with other aspects of the perceiver, such as endorsement of racial or sexual prejudice. Researchers might also examine the range of signals that various types of dress might give, both overall and for individual perceivers. For example, due to different experiences and associations, a perceiver who grew up in a poor rural community might see someone wearing a blazer and imply that this person is going to church, a wedding, or a funeral. On the other hand, a perceiver who grew up in an affluent neighborhood might instead imply that this person is going to work or attending a party.

Dress might also signal cognitive states as a function of target face/body. For example, formalwear might shift perceptions of threat most strongly for Black male targets who are very tall or muscular (Hester & Gray, 2018; Wilson et al., 2017; Yi, 2015). Or, conservative dress might shift perceptions of sexual interest the most for female targets whose face and body align with the perceivers’ ideal of attractiveness. Finally, perceivers might infer different intended behaviors from the same clothing based on body size. For example, basketball shorts or sweatpants might signal going to work out for targets who weigh less and dressing for comfort for targets who weigh more.

Another promising future direction might explore laypeople’s understanding of how clothing signals mental states and how they strategically use this information. As described in the story, some Black men don formalwear to actively mitigate associations with threat (Yi, 2015). And, although inferences of sexual interest from clothing sometimes lead to inaccurate inferences about the wearers’ mental state, people sometimes intentionally choose to wear clothing that signals lower or higher sexual interest. People’s use of dress-related strategies is not well-defined in the literature, and initial research on this topic would be best served by more qualitative and descriptive methods.

### Status

In 1577, Queen Elizabeth I of England proclaimed (edited for clarity):

“The briefe content of certayne Actes of Parliament, agaynst th’inordinate use of apparell.

None shall weare in his apparell any Silke of the colour of purpure;

Cloth of golde, Tissue;

But onlye the Kyng, Quene, and Kinges Mother, Chyldren, Brethré, Sisters, Uncles & Auntes;

and Except Dukes & Marquesses, to be may weare in dublets and sleevelesse cotes, Cloth of Gold, of Tissue, not exceadyng. v. if. the yarde, and Purpure in mantelles of the Garter” (The Briefe Content of Certayne Actes of Parliament, Agaynst Th’inordinate Use of Apparell, 1577)

This particular sumptuary law goes on for several more paragraphs, yoking dress to social class (which corresponds strongly with wealth) to such a degree that one’s social class could be easily and accurately inferred from one’s dress. Although this phenomenon plays out more subtly now, dress still plays a vital role in the signaling of status.

The *status* factor describes how perceivers use information provided by dress to make inferences about the wealth, social class, and power of the target. Although this factor might arguably be collapsed with social categories (e.g., a rich person versus a poor person), it merits its own factor simply because dress is specifically and intimately related to wealth and social class, and wealth is more consequentially linked to power and agency than most other kinds of social categorization. Clothing has functioned throughout history to explicitly signal wealth and social class. The questions perceivers are answering in this factor include “How much power does this person have?” “How many resources does this person have?” and “What is this person’s standing in society?”

#### Connections to Social Cognition Research

“Conspicuous consumption” is an economics term coined by Thorstein Veblen that describes the consumption of goods for the purpose of signaling wealth and/or social class (Veblen, 1912). So powerful is the ability of some goods to signal wealth that their value and demand are characterized by a Veblen curve—as market value increases, demand also increases (e.g., Bagwell & Bernheim, 1996; Sundie et al., 2010). The conspicuous nature of this consumption demands that the item be visible and understandable to perceivers, which is a clear characteristic of clothing, jewelry, and other aspects of dress.

Adam Smith, an 18th-century economist and philosopher, argued that the desire for status and wealth is near-universal and that being high status is associated with various benefits (Smith, 1776). Status anxiety is associated with negative health and societal outcomes (Layte & Whelan, 2014; Pickett & Wilkinson, 2010) and low subjective social status is associated with increased risk of cardiovascular disease, hypertension, diabetes, and lower well-being (Schneider, 2019; Tang et al., 2016). Low subjective social status appears to increase status-seeking behavior; for example, increased perceptions of income inequality correspond with increased conspicuous consumption (Du et al., 2022) and the frequency of internet searches for common status objects—most of which are articles of clothing—moderates the link between income inequality and risk-taking behavior (Payne et al., 2017).

Social identity can also correspond with lower subjective social status and incentivize specific groups to prioritize subjective status. For example, Black and Latine people in the United States spend a larger percentage of income to purchase visible luxury goods as a means of signaling status, an effect that is partly explained by actual disparities in income between groups (Charles et al., 2009). Furthermore, some American women report prioritizing wealth and status in their selection of men as mating partners (Buss & Schmitt, 1993; Li & Kenrick, 2006) and perceive men with more resources as more attractive (Wang et al., 2018), a reflection of long-standing social structures in which women’s primary means of social mobility was via marriage (though other work argues that these gendered preferences are weaker or nonexistent, at least in an American setting; Eastwick & Finkel, 2008). This lay understanding of women’s (and society’s) emphasis on high status for men may motivate men to engage in conspicuous consumption to improve their life and dating prospects (Griskevicius et al., 2007). Women’s preference for men signaling high status and men’s desire to consume high-status goods increased for participants primed with scarcity (Bradshaw et al., 2020).

Although perceivers might infer targets’ social status or wealth from their consumption, they are unlikely to do so very accurately from facial cues (Bjornsdottir et al., 2022; Bjornsdottir & Rule, 2017). Body-related cues, such as body mass index, might provide better information about social status and wealth due to gender-moderated “social gradients” of body mass index as a function of income and education (Claassen et al., 2019). Comparatively, target dress likely provides the most accurate information about social status and wealth.

#### Integration of Existing Dress Research

The inference of social status and wealth from dress manifests as early as 4 years of age. For example, Western children infer social status from the “new” versus “worn” state of objects such as blue jeans, mittens, and backpacks, and they associate this higher status with higher competence and health (Shutts et al., 2016).

Unsurprisingly, major differences in target dress (e.g., suit versus fast food uniform; Townsend & Levy, 1990) yield differences in impressions. However, perceivers also pick up on subtler cues. For example, perceivers rate men wearing custom-tailored suits, rather than off-the-rack suits, as more successful, salaried, and confident (Howlett et al., 2013). Brand names likely also make a difference. Notably, the effects of these dress-based status cues are persistent. Perceivers rated targets in “richer” clothing as more competent than targets in “poorer” clothing even when stimuli were presented for only 129 ms, when they were explicitly told that the clothing is not an accurate cue of competence, and when they were told to ignore clothing (Oh et al., 2020). Determinants of “status” may also differ for men and women. One study found that perceivers used formal dress to predict the status of male university faculty and staff, which contributed to accuracy because formal dress did correspond with their status. However, they did not use formal dress to predict the status of female university faculty and staff, and formal dress did not actually correspond with their status (Mast & Hall, 2004).

Finally, the fashion norms in higher-status locations exert greater influence on individuals in these locations than the norms in lower-status locations. For example, women moving from a higher- to a lower-status neighborhood are more likely to continue wearing the same type of shoe compared with women moving from a lower- to a higher-status neighborhood, who are conversely more likely to adopt the local shoe norms (Galak et al., 2016).

#### Future Directions

Research investigating the relation between dress and perceived status might start by mapping out variability in perceiver’s cultural knowledge and beliefs about the meaning of dress cues as conspicuous consumption, using both qualitative and quantitative approaches. Understandings of “rich” versus “poor” dress might vary considerably both between-culture and within-culture (as a function of socioeconomic status [SES] and other factors), as well as across time. One growing trend among high-SES consumers is the idea of “inconspicuous consumption,” in which classic conspicuous brands such as Coach, Rolex, and Hermès have been traded out for little-known boutique brands that only “in-the-know” consumers would recognize (Eckhardt et al., 2015). A related finding at the very high end of status is the “red sneakers effect,” which describes how nonconforming behavior (such as entering a luxury boutique or a board meeting wearing ripped denim, Vans, and a t-shirt) signals status (Bellezza et al., 2014). Finally, in some circles, vintage clothing is an indicator of status despite the used nature of the clothing, because vintage dress “requires a certain amount of cultural and economic capital” that necessitates both disposable income and free time (Veenstra & Kuipers, 2013, p. 356).

The meaning of dress-related status cues might also differ depending on other aspects of the target, such as their race, gender, and age. Men’s dress might determine perceived status more than women’s dress, and clear status cues such a bespoke suit or a nice watch might influence perceptions of Black or Latine targets more than perceptions of White targets because of greater deviation from baseline expectations. Similarly, perceivers might infer greater wealth from a Rolex watch on the wrist of a high schooler than they do from the same watch on the wrist of a middle-aged adult.

Furthermore, most investigations of status cues focus on specific clothing items or entire outfits that cue high or low status. Researchers might consider how pairing conspicuous dress objects with otherwise very casual and low-status outfits influences perceptions of status. This kind of nonchalant combination might be especially effective at signaling status, in line with the concept of *sprezzatura*, an Italian word referring to seemingly effortless (and thus desirable) aesthetic expression that requires a certain degree of confidence and cultural knowledge (see D’Angelo, 2018).

### Aesthetics

Early ideas about aesthetic appreciation identified the locus of beauty in objects themselves. However, by the eighteenth century, philosophers such as David Hume and Immanuel Kant adopted “eye of the beholder” arguments instead, placing beauty squarely in the minds of the perceivers (Sartwell, 2017):

Beauty is no quality in things themselves: It exists merely in the mind which contemplates them; and each mind perceives a different beauty. One person may even perceive deformity, where another is sensible of beauty; and every individual ought to acquiesce in his own sentiment, without pretending to regulate those of others. (David Hume, 1757, p. 230)

It is doubtless the case that perceivers express idiosyncratic preferences or “tastes” regarding aesthetics—people simply have favorite colors, patterns, shapes, designs, and so on.^1^ Aesthetic taste thus plays a unique and crucial role in how dress influences perceptions of others.

The *aesthetics* factor describes how perceivers’ preferences for basic elements of dress, such as color, fit, cut, texture, and so forth, influence impressions of targets. These preferences emerge from a mix of cultural influences and personal idiosyncrasies. Although this factor overlaps some with the first three factors, it crucially accounts for the fact that perceivers simply “like” or “dislike” certain kinds of outfits independent of any signaling of social category, cognitive state, or status. This is not unlike how people show preferences for art that are unexplained by the arts’ specific message or themes—preferred combinations of colors, shapes, textures, and patterns vary widely. We expect that this factor will demonstrate especially high amounts of perceiver and perceiver-by-target variability. The questions perceivers are answering in this factor include “Do I like what this person is wearing?” and “Does this person looks good in this kind of outfit?”

#### Connections to Social Cognition Research

Research on aesthetic elements in social cognition is limited in scope. Of the various aesthetic elements of dress, perhaps the most thoroughly researched topic is color. Much of the recent work on color in social cognition is grounded in evolutionary theory, attempting to draw parallels between human and nonhuman responses to specific colors. For example, red (as expressed in the reddening of the face or the skin) is characterized as a testosterone-based indicator in competitive interactions between males, and thus appears to signal dominance in some contexts (Elliot & Maier, 2014; Hill & Barton, 2005). Color-in-context theory integrates biology-based and context-based meanings of colors and states that the meaning of color varies depending on the motivations and mental states of perceivers (Elliot & Maier, 2012). In this way, aesthetic preferences for color might emerge from functional preferences. Although much of this evolutionary-based work focuses on psychological universals in how color influences cognition, it is essential to highlight cultural heterogeneity in color preferences—for example, British and Himba color preferences overlap very little and Himba color preferences display none of the “universal” patterns claimed elsewhere (C. Taylor et al., 2012).

#### Integration of Existing Dress Research

The most prominent research on aesthetic properties of dress concerns effects of red clothing on judgments of attractiveness and dominance (e.g., Elliot et al., 2013; Kayser et al., 2010). This work mostly draws on the same evolutionary-based theory discussed earlier, which associates red with testosterone and competition (Hill & Barton, 2005). The color black has also received some attention for its potential to increase perceived attractiveness (S. C. Roberts et al., 2010) and experienced dominance (Frank & Gilovich, 1988). These color effects do not seem to apply equally across gender dyads (S. C. Roberts et al., 2010). Finally, research suggests that the moderate matching of colors (not too similar, not too contrasting) might yield the most positive first impressions (Gray et al., 2014).

Other research on color concerns perceivers’ color preferences across time and place. A recent review of color preferences over time (Kodžoman et al., 2022) suggests that Western color preferences for dress have shown some stability over the past 100 years, with blue and red ranking higher in preference. However, the review points out some methodological limitations and finds that current color preferences for dress include yellow, pink, and black. Gender differences also emerge, with women rating yellow and white more highly than men (Kodžoman et al., 2022). Outside of scientific dress research, it is worth noting that preferences for dress clearly vary across time and place. The Western female-pink connection only emerged in the 1940s (Stamberg, 2014), and black, often viewed as a ubiquitous color in fashion, only became a common color in South Korean women’s clothing in the 1980s (Seok & Geum, 2012).

#### Future Directions

Although some work has been done on cultural and individual preferences for color, the current scientific understanding of perceivers’ aesthetic preferences is limited. Descriptive work might conduct a broad census of color, fit, cut, and style preferences with the aim of describing variability in these preferences across individuals and cultures. This type of approach can be valuable for understanding the types of predictors that might explain variability in preferences (Hester, Xie, & Hehman, 2021), which can inform subsequent work that considers how preferences vary systematically as a function of gender, race, age, and so on. Specific cultural influences might also emerge: for example, the association of specific colors or patterns with religion, politics, or nationality might shape perceiver preferences for these elements in dress.

Future work might also consider how perceiver preferences for specific aesthetic elements might be shaped by aspects of targets’ faces and bodies. A veritable mountain of magazine articles describes how certain colors of clothing look better on certain colors of skin; how different body types are flattered by specific cuts of clothing; how specific types of glasses frames suit specific face shapes; and how hairstyle should take into consideration the color, thickness, and natural tendencies of one’s hair. Researchers might measure the extent to which these popular recommendations influence and/or align with perceiver judgments.

In addition, little to no research has examined how dress influences perceptions of people with disabilities. On one hand, people with disabilities are not able to easily wear some articles of dress (Esmail et al., 2022). On the other hand, people with disabilities can make conscious decisions about specific design elements of physical aids such as wheelchairs, prosthetics, canes, and walkers. For example, one video commentator highlights the light-up casters and pink wheels of her new wheelchair (Joci Scott, 2021) and an online search for “wheelchair accessories” yields various joystick knobs, handle covers, foot slings, and other items that are clearly elements of dress. Future research might qualitatively survey people with disabilities to learn more about how physical aids are used as dress. Then, using this information, researchers might use variance partitioning to describe the extent to which these unique dress elements account for perceptions of people with disabilities.

Researchers might also consider the extent to which people’s aesthetic preferences for others’ dress matches their aesthetic preferences for their own dress. On one hand, there is ample evidence that people’s preferences for others tends to resemble their own preferences (Montoya et al., 2008). On the other hand, preferences for others’ dress appear to be strongly organized according to gender norms (and, to lesser degrees, other norms), such that people might prefer clear contrasts in aesthetics between men and women. Notably, this gendered concept of attractiveness appears to be loosening recently in both Western and some East Asian cultures as people’s gender concepts also become less rigid.

## Challenges and Recommendations

This working model of dress in person perception highlights many exciting directions for future research. However, the sprawling and complex nature of these research ideas presents both theoretical and methodological challenges. We discuss some of these challenges and offer recommendations.

### Theory

The sheer complexity of target dress—both on its own (e.g., full outfits comprised of several elements) and in combination with perceiver characteristics, target face/body, and target context—presents difficult theoretical challenges. When there are so many potentially interlocking parts, how can researchers accurately theorize about phenomena? Here, we find guidance in intersectional perspectives on psychology, which both warn against over-valuing parsimony in models (McCormick-Huhn et al., 2019; Settles et al., 2020; Warner et al., 2016) but also acknowledge the necessity of constraining conditions to conduct empirical research (Warner, 2008). Because the meaning of dress is so culturally variant, we recommend that research on dress draw broadly from outside of psychology to support hypotheses and justify the inclusion and exclusion of specific aspects of target dress (as well as other relevant variables). This sociohistorical approach to psychology has yielded insights into human cognition that do not readily emerge from a purely psychological perspective. This approach is most commonly seen in cross-cultural work examining differences in self-construal (Kitayama et al., 1997; Markus & Kitayama, 1991), racial categorization (Goh & McCue, 2021), tightness-looseness (Gelfand et al., 2011; Jackson et al., 2021), attitudes toward older adults (North & Fiske, 2015), and many others. Sociohistorical approaches can also be used to understand processes in more constrained cultural settings, as is the case in work examining social dominance orientation (Ho et al., 2012; Sidanius et al., 1994) and hypodescent (Cooley et al., 2018; Ho et al., 2011; Krosch et al., 2013). Given the breadth of research examining fashion, a similarly broad perspective is essential for moving toward a comprehensive model of target dress in person perception.

Another recommendation for navigating the complexity and scope of target dress is to embrace descriptive research as a necessary first step toward predictive models and theories. Without closely observing myriad fauna and flora, Charles Darwin would not have been able to develop a theory of evolution. In the same vein, we suggest that the simple observation and description of perceivers’ judgments of target dress, as well as the “lenses” through which they understand target dress, is necessary before trying to formulate comprehensive theories of person perception that include target dress.

### Method

Perhaps the biggest methodological challenge for studying dress is creating adequate stimulus sets for perceivers to judge. Of particular note is the issue of separating effects of target body from target dress—ideally, a stimulus set would fill in a “matrix” in which many types of bodies are depicted wearing many types of clothing. For this reason, the development of large, coded sets of stimuli featuring full crosses between body and clothing (and the inclusion of photographs with and without faces) would facilitate future research. These types of databases for facial stimuli have been of great utility (e.g., Ma et al., 2015; Saribay et al., 2018). However, the challenges of forming such databases for target dress are considerably higher. More importantly, even if generalizable and representative dress datasets were ever created, these datasets might quickly become dated due to the quickly shifting styles and meanings of dress. This challenge is not present with biologically constrained stimuli, such as faces. It might be the case that investing time and resources into creating a representative clothing database would not be worthwhile.

For this reason, researchers may need to develop their own specific stimulus sets—motivated specifically by their research questions—by drawing on existing photographs and using photo editing techniques. One promising direction that might help “solve” the conundrum of dress stimuli is the development of Generative Adversarial Networks that specifically generate novel images of clothing (Ak et al., 2019; Cheng, 2020/2021). With some adjustments, this approach might have the potential to generate sets of realistic-looking images across target body type, target dress, and target face. Although this type of computer-generated dataset might not be fully representative of current styles of clothing, it may nevertheless allow for a more wide-ranging set of dress stimuli to be created at little cost. Researchers have also created fairly realistic-looking stimulus sets that vary face, body, and clothing by using photo-editing software to swap faces onto bodies and match the luminance of the skin (Connor et al., 2023).

Once researchers have stimulus sets in place, within-subject designs should be used whenever feasible for two reasons. First, these designs offset the otherwise substantial costs of collecting enough data to example more complex issues. Second, these designs can often facilitate the partitioning of variance into different sources (Hehman et al., 2019; Kenny et al., 2006). For example, research has described the extent to which variability in impressions of faces is broadly caused by perceiver characteristics, target characteristics, or interactions between perceiver and target characteristics (Hehman et al., 2017; Hönekopp, 2006; Xie et al., 2019, 2021). This approach is useful for describing the “landscape” of variance when exact predictors of interest are unclear or too numerous to measure and model.

Taking to heart the value of descriptive work, researchers might also consider the use of qualitative methods to collect rich, nuanced information about how impressions might emerge from combinations of perceiver, target, and contextual factors. In the same way that qualitative and mixed methods approaches promote understanding of complex phenomena such as intersectional stereotyping and discrimination (Cole, 2009; Else-Quest & Hyde, 2016), they may also promote understanding of the role of target dress in person perception.

Researchers must also consider the cultural background of both the perceivers and the targets. The primary constraint on generality in research on dress and person perception is that the findings synthesized in this review predominantly rely on ratings of White American perceivers presented with White American targets. First, regardless of the perceivers and targets sampled, researchers should carefully assess and accurately portray the generalizability of their findings. Second, to achieve greater generalizability, researchers might sample broadly from various locations; create more representative stimulus sets, especially when it is clearly relevant to the present hypotheses; incorporate sociohistorical approaches to make meaningful predictions about the meaning of dress in specific cultural settings; and form research teams that represent a broader range of cultural backgrounds. To the latter two points, the authors of this paper are North American cis straight men—the first is half-Filipino and half-White, and the second is White. Both are academic experts on person perception and stereotyping, and the first author is additionally (a) someone who uses an intersectional lens to examine psychological phenomena and (b) a clothing/style enthusiast in general. However, given the singular nature of identity, many other lived experiences of the importance of dress are only indirectly accessible to the authors. Considering this positionality is important, particularly given that much of the cited research in this paper is led by cis White American men, particularly the older work in person perception and social cognition.

Finally, to better capture the extensive cultural variability in perceivers’ understandings of target dress, a multi-lab collaborative approach might be necessary to collect a globally diverse dataset with accurately translated instructions and questions. This step would likely come after the landscape of target dress and person perception has been sufficiently described, as multi-lab collaborations are time-intensive and expensive. However, as illustrated by the Psychological Science Accelerator (Jones et al., 2021; Moshontz et al., 2018), the dividends paid by this type of collaboration are substantial—for example, one project in this vein yielded over 11 million ratings of 120 faces across 45 countries (Jones et al., 2021) as a means of evaluating heterogeneity in the factor structure of face perception across cultures.

## Conclusion

Legendary costume designer Edith Head once stated that “[y]ou can have anything you want in life, if you dress for it.” This quote captures the undeniable importance of dress in shaping people’s impressions of others. And yet, dress is notably absent from decades of theorizing about first impressions, which has emphasized the role of faces and bodies. The result is a mismatch between how first impressions unfold in the lab—a parade of floating heads and gray-white backgrounds—and how they unfold in the world—a whirling of faces, fabrics, bodies, and baubles. Social psychology is past due in recognizing and accounting for the essential role of target dress in person perception. Doing so might require a shift in priorities, eschewing the traditional emphasis on parsimony and psychological universals in favor of necessary complexity and cultural variability. This process will require some trial and error—not unlike putting together an outfit for a job interview or a first date—but the effort will be well worth it.

## Notes

1. William James had a particular fondness for polka dot ties, which were notably unfashionable at the time (see Watson, 2004).
